# Physical activity and the environment: conceptual review and framework for intervention research

**DOI:** 10.1186/s12966-017-0610-z

**Published:** 2017-11-15

**Authors:** Jenna Panter, Cornelia Guell, Rick Prins, David Ogilvie

**Affiliations:** MRC Epidemiology Unit & Centre for Diet and Activity Research (CEDAR), Box 285, School of Clinical Medicine, University of Cambridge, Cambridge, CB2 0SR UK

**Keywords:** Evaluation, Intervention, Environment, Physical activity, Systematic review

## Abstract

**Background:**

Changing the physical environment is one way to promote physical activity and improve health, but evidence on intervention effectiveness is mixed. The theoretical perspectives and conceptual issues discussed or used in evaluative studies and related literature may contribute to these inconsistencies. We aimed to advance the intervention research agenda by systematically searching for and synthesising the literature pertaining to these wider conceptual issues.

**Methods:**

We searched for editorials, commentaries, reviews, or primary qualitative or quantitative studies in multiple disciplines by electronic searches of key databases (MEDLINE and MEDLINE In-Process, Web of Science, Cochrane Reviews, ProQuest for dissertations, Health Evidence, EPPI-Centre, TRID and NICE) and snowballing. We extracted theoretical and conceptual material and used thematic analysis in an in-depth, configurative narrative approach to synthesis.

**Results:**

Our initial searches identified 2760 potential sources from fields including public health, sociology, behavioural science and transport, of which 104 were included. By first separating out and then drawing together this material, we produced a synthesis that identified five high-level conceptual themes: one concerning outcomes (physical activity as a behaviour and a socially embedded practice), one concerning exposures (environmental interventions as structural changes) and three concerning how interventions bring about their effects (the importance of social and physical context; (un) observable mechanisms linking interventions and changes in physical activity; and interventions as events in complex systems). These themes are inter-related but have rarely been considered together in the disparate literatures. Drawing on these insights, we present a more generalisable way of thinking about how environmental interventions work which could be used in future evaluation studies.

**Conclusions:**

Environmental and policy interventions are socially embedded and operate within a system. Evaluators should acknowledge this, and the philosophical perspective taken in their evaluation. Across disciplinary fields, future studies should seek to understand how interventions work through considering these systems, the context in which interventions take place, and the (un) observable mechanisms that may operate. This will help ensure that findings can be more easily interpreted and widely applied by policymakers. We hope that highlighting these conceptual issues will help others to interpret and improve upon a somewhat contested evidence base.

**Electronic supplementary material:**

The online version of this article (doi: 10.1186/s12966-017-0610-z) contains supplementary material, which is available to authorized users.

## Background

Physical inactivity is a major risk factor for non-communicable disease [1]. It has an effect on health similar to that of smoking or obesity, and has been estimated to account for 9% of global mortality [2]. As a result, physical activity promotion is the subject of numerous government action plans [3]. Reviews of the effectiveness of interventions to promote physical activity have largely focussed on specific behaviours (such as walking or cycling [4]), contexts (such as workplaces [5]), or ways to intervene (such as brief advice [6] or changing the environment [7]). Addressing the latter by tackling the points in the social and physical systems that generate and sustain inactivity may be one way to make physical activity easier. Environmental approaches may include such interventions as the construction of new walking and cycling routes or the development of new green spaces.

Systematic reviews have the explicit aim of critically appraising and synthesising existing research on a topic [8]. Traditionally they have focussed on summarising evidence on the effectiveness of interventions. In this area, many reviews have concluded that the evidence on effectiveness is limited and the findings from the best available studies are sometimes mixed [9, 10]. For example, Mayne and colleagues found inconsistent results even between studies with the strongest designs [9]. Bringing together evidence on the effectiveness on environmental interventions is challenging for several reasons. Interventions are heterogeneous in terms of their content, delivery and context, and are often implemented by policy makers or practitioners with limited opportunities for planned evaluation [10]. Where evaluations do occur, these may be conducted by researchers from a variety of disciplines or by multidisciplinary teams [11]. Studies may report impacts on different forms of physical activity, in different groups of people, and over different time periods. Different methods and data sources may be used, and authors may have different perspectives about what exactly is being evaluated and what might constitute evidence of success. These different views may be amplified in multidisciplinary teams, and some of these differences are predicated on conflicting epistemological perspectives which may not always be acknowledged. In many reviews of effectiveness these perspectives are not considered, and the analysis may focus on a particular outcome or type of study design. Another key challenge for synthesis is that authors do not clearly describe how interventions are expected to work – the ‘intervention theory’ –or investigate how they work in practice to bring about their effects [10]. Describing and testing plausible mechanisms of this kind is important for strengthening causal inference and the utility of evaluative studies for policy and practice [12].

Eliciting and synthesising conceptual thinking from multiple disciplinary perspectives, overarching theories and more generalisable processes could contribute to a better understanding of the results of existing studies, especially when these are contradictory [11], and to the design of more useful intervention studies in future. In this paper we therefore aim to draw out and synthesise relevant conceptual issues raised from different disciplinary and epistemological perspectives in evaluative studies and the wider literature, and apply these to our thinking about how changes in the external physical environment might act to promote physical activity. In doing so, we identify areas of common ground between disciplines, use the most relevant perspectives to describe a more generalisable framework for thinking about how environmental interventions may bring about their effects, and describe the implications for future intervention research. This initial conceptual analysis precedes a detailed consideration of the empirical evidence for potential mechanisms to understand the outcomes of particular interventions, which forms the topic of a second – separate – review not reported in this paper.

## Methods

### Overall approach

We adopted a systematic and transparent approach to identifying relevant conceptual material, used a qualitative approach to synthesis and presented our results narratively, as outlined in relevant guidance on synthesis in systematic reviews [13, 14]. It seemed clear that this review would require openness to transcending disciplinary and epistemological boundaries in order to be able to abstract and generalise from diverse types of specific evidence [15]. As a result, our approach was pragmatic, pluralistic and interpretive and this approach is echoed in other meta-narrative reviews (e.g. [16]).

### Search strategy

As we sought diverse evidence from a variety of disciplines and judged that it would be impractical to search comprehensively for material, we used an expansive search rather than an exhaustive one [8, 17]. We used several approaches for searching, each being designed to complement the strengths and limitations of the others. We searched eight electronic databases encompassing a variety of disciplines (MEDLINE and MEDLINE In-Process, Web of Science, Cochrane Reviews, ProQuest for dissertations, Health Evidence, EPPI-Centre, TRID and NICE). We used search terms to identify reviews and other sources likely to provide conceptual and theoretical material relating to physical activity and the environment and applied these to the titles and abstracts of papers in the databases. Full details of the search terms are shown in Table 1. These terms were adapted from those used in a previous review of reviews in this topic area [18] – which included papers from a variety of disciplines including transport, urban planning and public health – with the addition of terms to identify conceptual and theoretical material following recommendations of Booth and colleagues [19]. We acknowledge that this is a limited set of search terms, but we sought a balance between increasing the number of search terms and the likely number of relevant results. Pilot testing confirmed that three ‘seed’ papers we considered to contain relevant conceptual material were captured using this strategy [11, 20, 21]. We also searched the reference list of each included paper to identify recent relevant reviews and key sources for cited theoretical works where required, and added relevant papers from the authors’ personal collections. We applied no country, language or date restrictions.

**Table 1:** Search terms

Study design	Physical activity	Environment
concept* OR theor* OR framework OR review* OR systematic OR synthesis OR summary	physical activity OR exercise OR walking OR bicycling OR cycling	environ*

* denotes wildcard symbol

### Inclusion criteria

Given that different disciplines may use terminology in different ways [8], we took an inclusive approach to selection. Sources could be editorials, commentaries, reviews, or primary qualitative or quantitative studies. However, grey literature was not included because we judged this would be unlikely to inform additional key concepts or theories. Sources had to contain theoretical material relating to the ways in which physical activity behaviour may or may not change in response to environmental change. They could describe an application or critique of a published theory, provide information on a theorised model of change which may have been empirically tested, present a logic model, or discuss theories in the context of results from an intervention study. Sources did not have to cite a published theory to contribute conceptual material, whereas *only* citing a theory, model or perspective was insufficient for inclusion. Any material defined as a theory, concept, framework or model was included (see Table 2). After obviously irrelevant references were removed, one reviewer (JP) assessed all remaining titles and abstracts for inclusion.

**Table 2:** Definitions of key terms used in the review (From Glanz and Rimmer [32])

Term	Definition
Theory	A set of inter-related concepts, definitions or propositions that present a systematic view of the events or situations by specifying relations among variables to explain and predict events or situations. It is general and broadly applicable
Concept	These are the building blocks of a theory
Framework	A structure for presenting concepts, without necessarily preserving the inter-relationships between them
Model	Similar to a theory, a generalised or hypothetical description used to analyse or explain something

### Extraction and coding of conceptual material

We extracted theoretical or conceptual descriptions, interpretations or conclusions offered in each source into a spreadsheet and organised these using emerging and iterative descriptive codes. We took a pluralistic approach, extracting abstract explanations, theory-informed interpretations and the less visible (implicit) occurrences of theory submerged within each text [22]. Two reviewers (CG and RP) corroborated the extraction and interpretation of texts through repeated reading and discussions.

### Interpretive thematic synthesis

We used a stepwise thematic approach for the synthesis [8]. This involved an iterative process of reading and re-reading of material as well as the independent identification of themes exploring the ways in which environmental changes were thought to influence behaviour, the disciplinary perspectives and the approaches used. Where key theoretical sources were cited in the included papers, these were read and interpreted in the context of the original citation. We merged the longlist of initial descriptive codes into overarching themes and contextualised and interpreted our results using the included papers (denoted with ^S^) alongside the wider literature, including intervention studies which did not result directly from the searches. We completed our electronic searches in April 2015 and followed these up in the wider literature in June 2015. All authors contributed to the interpretation and synthesis.

We chose not to identify an a priori framework for the analysis, as this would have shaped the way evidence was collected and appraised [23]. However, the authors’ own judgements and disciplinary perspectives inevitably played a role in shaping the synthesis. These were informed by the authors’ training in a diverse range of social and biomedical sciences (anthropology, environmental sciences, human movement sciences and public health medicine), as well as all authors’ experience and appreciation of the use of both quantitative and qualitative methods, and over 30 person-years of experience in interdisciplinary research in the topic area.

## Results and discussion

### Overview of included sources

The electronic searches provided 2760 unique records, of which 77 met the inclusion criteria for full text screening (38 were reviews of empirical studies and 39 were conceptual papers). Not all reviews contained relevant conceptual material or evaluative studies. Snowball searching from these provided a further 27 relevant papers, bringing the total to 104. The full list of included sources is provided in Additional file 1 ^S1–104^.

Although all included papers provided conceptual insights, they were predominantly focused on either the outcome of interest (the promotion of physical activity, e.g.^S83^); the exposure (the role of the environment in shaping health, e.g.^S23^); or how interventions might work (the role of environmental changes in shaping physical activity in particular, e.g.^S76^). Authors’ disciplinary and epistemological perspectives were demonstrated through the language, methods and approaches used, as well as the journal in which papers were published. Sources came from fields such as economics, epidemiology, exercise science, sociology, psychology, public health, and urban planning but many were interdisciplinary. Many papers cited published theories in their introduction as having shaped their perspective (e.g some cited a specific model and described how a variety of interpersonal, social and physical characteristics explain behaviour) but few described how changes in environments might lead to changes in physical activity behaviour.

### Emerging themes

In the course of the analysis, five overarching inter-related themes emerged: one concerning outcomes (*conceptualisations of physical activity*), one concerning exposures (*environmental interventions as structural changes*), and three concerning how interventions bring about their effects (*Context may alter the success of an intervention; mechanisms may be observable or unobservable; and interventions as events in complex systems*). Table 3 gives an overview of the narrative we derived from specific conceptual issues and how they relate to these five themes. The implications for future research are discussed within each theme. The references cited under each theme are examples of source papers in which the themes are discussed, rather than an exhaustive list. We do not aim to describe particular conceptualisations as ‘right’ or ‘wrong’, but to lay out the different approaches used and understand the strengths and limitations of each. In reviewing the codes and themes, it was apparent that the perspective and specific approach taken was highly dependent on the philosophy of science in that discipline.

**Table 3:** Summary of overall narrative and relationships of specific points to overarching themes

Themes					
Conceptual points for discussion	1. Conceptualisations of physical activity	2. Environmental interventions as changes in structure	3. Context may alter the success of an intervention	4. Mechanisms may be observable or unobservable	5. Understanding interventions as events in complex systems
Physical activity means different things to different disciplines…					
movements (exercise physiology)	✓				
types of activity (behaviours)	✓				
a collection of activities (practices)	✓				
Influences on physical activity are viewed differently in different disciplines…					
social influences (psychology) cf. socially embedded (anthropology, practices)	✓	✓		✓	✓
physical influences (could be as a context)			✓		
Physical activity behaviour and its influences are complex and inter-related	✓				
Definition of the environment includes attributes of the social and physical environment		✓		✓	✓
Interactions between people and environment…					
people as agents		✓			
environments as structural constraint		✓			
Effectiveness of interventions differs…					
and could be altered by social or physical environments			✓		
or trigger different processes				✓	
Methods for assessing mechanisms differ…					
Observable and measurable: quantitative methods (mediation or moderation) or qualitative methods				✓	✓
Unobservable and unmeasurable: qualitative methods				✓	✓
Important to acknowledge that ..					
feedback loops or reciprocal pathways (interactions between people and structure) operate		✓		✓	✓
long causal pathways are non-linear		✓		✓	✓

### Conceptualisations of physical activity

Authors reported broad conceptualisations of (changes in) physical activity and different perspectives on the social connectedness of activity, which in part reflected the foundations of different research fields.

#### (a) Physical activity as movements, behaviours and practices

Physical activity is often defined mechanistically within a biomedical perspective as ‘any bodily movement produced by skeletal muscles that require energy expenditure’ [1], but in the reviewed papers – as in physical activity promotion in general – behaviours or actions such as walking or cycling, rather than movements, were the targets of interventions. Laitakari and Miilunpalo highlighted the need for a pragmatic and wide concept of physical activity which includes a combination of several activities or actions, including sports and transport-related activity, not a single behaviour^S56^. Similarly in the sociology literature, studies often conceptualised activity as bundles of actions or behaviours and referred to these as ‘practices’, such as active commuting - sets of inter-related and interconnected activities, rather than discrete behaviours^S15, 75^.

Environmental changes such as the creation of new green spaces may target particular groups or types of activities, but may also encourage a wider range of activities in the general population. These were often reflected in the physical activity outcomes used in evaluations, which included direct observations of the number of users of a space, those who were physically active, or those who were engaged in specific activities. Assessments of the volume of, or time spent, walking, cycling or in total physical activity through population-based surveys were less common^S48^. Other authors described how spaces may be important places to meet rather than specifically to engage in sport^S1^, illustrating how physical activity may be combined with other, more sedentary activities. This may complicate efforts to evaluate the impacts of interventions on ‘physical activity’.

#### (b) Physical activity as a socially shaped behaviour or practice

Authors also reported individual and collective perspectives on the social connectedness of activity. Sallis and colleagues^S88^ observed that individually-oriented perspectives have dominated physical activity research. For example, the widely-used Theory of Planned Behaviour^S3^ suggests that social norms influence behaviours and that an individual’s attitudes to cycling, for example, might be shaped by their perceptions of its social acceptability as an unusual or a common activity. Similarly, the COM-B model includes capability (C), opportunity (O) and motivation (M) as determinants of behaviour, with opportunity encapsulating both physical access and social acceptability^S65^. Authors also suggested that others’ behaviour can influence an individual’s behaviour as a cue to action^S83^, enforce patterns of social control, or place constraints on individual choice through interpersonal relationships^S64, 75^.

In contrast, many sociologists and community psychologists viewed behaviour from a societal perspective. Here, the focus is not on an individual’s perception of social norms but on an understanding of physical activities as social phenomena or practices^S33, 75,^ which are shared, learned, and influenced by others and where people live^S33^. Authors described how repeated engagement led to a practice becoming embodied as participants began to identify themselves as ‘cyclists’ or ‘runners’^S75^ [24]. Shove and colleagues distilled practice into three interconnected components: meanings, which reflect shared understandings; materials; and competencies, which refer to embodied knowledge^S91^.

In other words, the notion of the social connectedness of physical activity has been operationalised in two different paradigms, each of which has validity. They share a core of understanding of behaviour, and the components of practice theory (competencies, materials, and meanings) are echoed in the COM-B model (capability, opportunities and motivation).

#### Implications

There are different but complementary approaches to conceptualising activity (movements, behaviours and practices) and this suggests that the definition of physical activity used by evaluators should not be limited according to a particular method or a discrete, narrowly defined set of behaviours or theory. Doing so may lead to inadequate capture of important outcomes and unintended consequences, and thereby produce misleading results. Working in multidisciplinary research teams is recommended and although authors have highlighted the challenges of this way of working^S76^, some of these may be resolved by actively and openly engaging with and understanding different approaches and acknowledging the different epistemological perspectives used.

Perspectives from psychology (e.g. the COM-B model) and social science (e.g. social practice theory) highlight interactions between components that determine behaviour. This suggests that the success of environmental interventions may depend not only on changing aspects represented by ‘materials’ or ‘opportunities’, but also on the links to other components. Take for example the construction of a new cycle path, segregated from motor traffic but running adjacent to a road. Using a social practice lens, the introduction of this new ‘material’ and its success in changing cycling practices might be enhanced if it also changed the meanings (practical benefits such as the convenience of cycling, or wider social conventions about cycling to work) or competencies of cycling (such as providing a way to avoid dangerous roads, the knowledge about which destinations it serves, or how to best use it safely to avoid conflict with other cyclists). More careful consideration of the design, implementation and promotion of the cycle path, or the adoption of a more multi-layered intervention strategy, might achieve greater effectiveness by maximising the synergy between ‘meanings’ and ‘competencies’. For evaluators this might mean an explicit consideration of how these other aspects changed as a result of the new ‘material’ of the cycle path. This way of thinking about interventions and their evaluation could lead to more relevant and powerful findings for policy and practice. Although some evaluations of environmental interventions have taken an avowedly sociological perspective [25], we are not aware of any that have specifically adopted a social practice theoretical perspective and assessed social practice as an outcome.

### Environmental interventions as changes in structure

Authors described different perspectives on how changes in the environment may ‘work’ to change behaviour^S35, 53, 76^. These reflected underlying differences in the perceived importance of human agency (“capability of individuals to do something”, (p.9) and of structure (“rules, resources or sets of relations, organised in systems”) (p.25)^S34^. Here, rules could be social norms or expected behaviours and resources could be money, social support or physical accessibility.

Many authors described conceptual positions in which individual agency dominates over structural influences, in that individuals are responsible for their actions and structural conditions have an implicitly secondary role^S2, 14, 29, 76^; these are described in formal theories such as the Theory of Planned Behaviour^S3^ and rational choice theories^S29^ from the fields of psychology and economics respectively. From this perspective, structural conditions (the ‘environment’) tend to be considered only insofar as they constrain or facilitate behaviour, often in terms of the alternative behaviours, their costs, and the rules and resources that constrain them. While researchers from all disciplines may agree that structural environmental conditions constrain or facilitate choices, and that changing the environment may therefore encourage physical activity by increasing the opportunities for it, other authors suggested that approaches which explicitly embrace the duality of agency and social structure, as outlined in structuration theory, might enable a better understanding of human behaviour^S14, 86^. Numerous formal theories or models such as social cognitive theory^S10^, field theory^S57^ and the socio-ecological model of health^S96^ emphasise this dynamic relationship. Lewin stresses that the person and their environment should be considered as one constellation of interdependent factors including time, place and social surroundings^S57^ –a formulation that echoes the triad of ‘person’, ‘place’ and ‘time’ traditionally considered in descriptive epidemiology, albeit often not as synergistic factors.

Some authors went beyond describing the mutually reinforcing constructs of agency and structure to acknowledge ways in which individuals shaped their environments more generally^S14, 86^, in some cases providing specific examples of how this might work. It was suggested that as well as physical attributes, environments possess social and cultural meanings^S1, 13, 46, 76, 83^ “characterised by an inherently dialectical relationship between physical reality and metaphoric and social construction”^S1^. From this perspective, descriptions of the environment are socially constructed and based on actions, perceptions, and interpretations. For example, assessments about whether a road is safe for cycling may depend on the volume of traffic; how other cyclists, pedestrians, and drivers use the space; and other cyclists’ reported preferences or behaviour, perceptions of risk aversion or media stories^S58^. As a result, attributes and perceptions of the physical and social environments may be intertwined.

#### Implications

In this field, the notions of agency and structure have often been applied (implicitly or explicitly) in a somewhat individualistic, positivist paradigm in which individual agency dominates over structural influences. However, if environmental interventions are likely to be characterised by the interplay between agency and structure, we suggest that evaluators should seek to assess these interactions to assist in the interpretation of their findings. They should also seek to understand people’s judgements and interpretations of the environment and acknowledge the reciprocal and dynamic relationships between people and places. For example, changes in the perceived supportiveness of the environment might form an important part of the process of changing population physical activity patterns^S14^. These changes in perceptions may take time^S58, 76^ and involve perceptions of the suitability of the environment for physical activity, the process by which the environment has changed and how others react^S14^. There are several ways in which such changes might be assessed, using both qualitative and quantitative methods.

### Context may alter the success of an intervention

Many authors described the importance of context in determining physical activity and the effectiveness of interventions to promote it. Here we define context as: “the physical, social, political and/or organisational setting in which an intervention was evaluated, or in which it is to be implemented” (p.119)^S87^. This broad definition seemed most appropriate as it embraced a range of types of evidence.

Different contexts were most often described as altering or moderating the relationship between the environment and physical activity^S2, 53^ and the success of interventions^S101^, including explaining a lack of success or smaller than expected effects in evaluative studies^S18, 48^. Context was considered at multiple scales, ranging from the characteristics of the individual or their immediate social group to the attributes of a city, region or country (e.g. topography or climate)^S4^. Individual life-stage and circumstances were described as contexts^S23, 27, 97^ that may play a stronger role at some times than at others^S4^. For example, changes to the environment might be most widely accepted among individuals who have already changed their behaviour and maintained it for a while; or among those already trying to change^S27^, who may look more favourably upon society’s actions to support their intentions. Environmental features may also be more or less important for different population groups, such as the elderly or working adults who tend to spend different amounts of time in their neighbourhoods and to have different lifestyles and functional capabilities^S23, 97^. For example, Diez-Roux suggests that the aesthetic quality and safety of the local area may be especially relevant for the elderly, who may derive most of their activity from walking in a relatively restricted geographic area^S23^, whereas such local features may be less relevant to working adults who spend less time around their homes. Local physical environmental contexts may also constrain or enhance the success of an intervention, for example if the use of new outdoor exercise equipment is constrained by a high level of crime or fear of crime in the neighbourhood^S101^.

Several authors described, and some contrasted, different approaches to analysing and understanding context underpinned by different disciplinary paradigms^S23, 29, 53, 89^. Epidemiologists often attempt to account for context by including variables such as income or social class in analyses e.g. by including variables such as income or social class as covariates in analyses and attempt to account (‘control’) for context. Some authors have reflected on this positivistic approach, suggesting that it strips these constructs of their social context and meaning^S21, S35, S94.^ These reflections have come both from within epidemiology and related disciplines such as public health, as well as from social sciences that take a more explicitly interpretivist approach. Sociologists such as Frohlich argue that inter- and intra-personal context cannot be easily isolated or quantified and that context is created by relationships between people and places^S33^. Other authors suggest that individuals have different ways of seeing the world (‘constructs’) which are a function of their own personalities and cognitions^S97^. In a similar way, Bourdieu suggests that people live by a personal set of cognitive and somatic (embodied) dispositions, termed habitus. These dispositions are acquired from collective experiences and understandings, shared and learned within a particular socio-cultural environment or context, considered normal, possible or idiosyncratic, and explained as ‘second nature’ (p.56)^S15^. In this way, habitus and personal constructs are ways of viewing context, in which the combination of an individual’s own experience, past behaviour and observations shape the lens through which the world is viewed.

Some authors suggested that different contexts lead to different outcomes because different processes and responses, or mechanisms, are triggered^S76, 86^, drawing on principles of realist evaluation. Mechanisms have been defined as the “underlying entities, processes, or structures which operate in particular contexts, to generate outcomes of interest” (p.368)^S7^. In the papers reviewed, contexts were conceptualised through a mixture of interpersonal, social and physical lenses. For example, routes for cycling may be viewed differently by inexperienced cyclists wary of conflict with motor vehicles on the one hand, and experienced cyclists on the other^S75^. Potential intervention mechanisms such as (changes in) concerns about traffic safety and fear and risk of being involved in an accident may therefore differ between these groups. Similarly, Abraham and colleagues suggest that landscapes are perceived and used differently by various social groups because of links to meaning, identity, attachment, belonging, memory, and history^S1^. These meanings or attachments could also be described as social or collective contexts or mechanisms: the historical context of an area could lead to new or improved environments not being used because of a lack of a sense of belonging or attachment, for example causing local people to perceive new facilities as being ‘not for us’ [26].

Two authors also highlighted that environmental interventions themselves and the ways they work might be context-specific^S76, S101^. In the case of new walking and cycling routes in the Connect2 project, Ogilvie and colleagues^S76^ noted that interventions of this kind were context-specific and that it might not be appropriate to directly culminate or compare results between different contexts. Watts and colleagues^S101^ also noted that the generalisability of environmental interventions to promote physical activity between places might depend on contextual environmental factors, the causal pathways between the environment and physical activity and the interaction between environmental factors.

Some authors argued that supportive environments may be necessary but not sufficient for behaviour change because of the role of individual or personal contexts^S35^. Realist researchers have also noted that mechanisms might not fire as a ‘dual on/off switch’, particularly where human volition is involved, corroborating the argument that favourable contextual conditions may not be enough to ensure an effect^S22^. To return to the example of a new cycle path, the varying degrees to which the new route may be convenient, or an individual may feel confident or safe in using it, may lead to the development of changes in cycling behaviours or practices in a more gradual or graded way. These observations suggest that while environmental changes may help to facilitate changes in physical activity at the population level, they may not be sufficient on their own.

#### Implications

Quantitative and qualitative methods, with their respective strengths and limitations, may both play important roles in understanding contexts and assessing their combined importance in determining the success of an intervention. Describing and assessing contexts using the most appropriate method for a given investigation is the key to ensuring the results of a study in one context can be understood and interpreted in a range of other contexts, which will help in understanding the generalisability (and limits to generalisability) of study findings.

### Mechanisms may be observable or unobservable

Many authors highlighted the need to describe the theoretical process of change of interventions and collect evidence for different hypothesised mechanisms^S23, 53, 76^. Although there may be wide agreement that evidence for causation is strengthened when there is evidence for both outcomes and plausible mechanisms of interventions, the concept and description of, and evidence for, mechanisms was reported differently by authors from different disciplines.

The definition of a mechanism of change in behaviour as a ‘process’ invites a discussion as to whether this process is conscious or unconscious. Conscious ‘reasoning’ is a reflective and cognitive process and this has been the approach to analysing mechanisms typically used in health psychology (for example, in models such as the Theory of Planned Behaviour^S3^). Some authors have explicitly suggested that conscious decision-making might be separated into two parts: practical consciousness, which refers to what is done through implicit knowledge and is difficult to express in words; and discursive consciousness, which refers to the explanation for behaviour when asked or a post-hoc rationalisation of it^S34^. The practical consciousness of a tacit understanding of how (or how not) to behave is also described by Bourdieu’s notion of habitus^S15^, whereby people live by a personal set of cognitive and somatic (embodied) dispositions.

In the sources reviewed, conscious decision-making was mentioned but the two parts were rarely distinguished. Unconscious mechanisms involving no or little cognitive processing received less attention than conscious ones, but were described in relation to the individual in terms of emotional responses and habitual processes, as well as in relation to collective or societal mechanisms in the form of modelling and mimicry^S55, 83^. Many authors referred to dual-process models of behaviour that combine such unconscious (semi-automatic) processes with more conscious (rational) processes in relation to goals and analytical decision-making^S1, 55, 65^. For example, Bauman and colleagues^S12^ suggested that installing a footpath could act as a cue to physical activity, and that knowledge of its history and perceptions of the environmental conditions along it could mediate its effects on behaviour. Whether these processes are explicitly conscious is subject to debate, particularly as recent studies investigating such mechanisms have found little evidence of changes in perceptions of environmental conditions as mediators [27]. In their review, Blacksher and colleagues suggested that both individual and collective mechanisms may be operating and may involve several intermediate steps, including perceptions of the process by which the environment changes, the reactions of others, deliberation over how to respond in light of the broader social context and competing personal goals^S14^. Different interventions might also lead to similar ‘downstream’ changes in reasoning (mechanisms). For example, a new segregated cycle path or new lighting along an existing path might both result in a change in reasoning to the effect that cycling is less risky, but this might come about through perceptions of greater physical segregation from traffic on the one hand or greater conspicuity on the other.

#### Implications

As in the previous themes, we would argue that quantitative and qualitative methods both have important roles to play in elucidating mechanisms of interventions. Tacit mechanisms such as role modelling and mimicry may be difficult to measure in questionnaires, or even in qualitative interviews alone. Ethnographic methods such as detailed observations and interviews [28] could be used both to observe and to infer such ‘unconscious mechanisms’ and to give people the opportunity to reflect on or rationalise their experiences, and this suggests that some mechanisms may be more observable to some disciplines than others. Societal contexts and mechanisms are likely to be particularly important for environmental interventions and could be investigated using interviews and observations alongside media analysis or documentary analysis, providing an important counterbalance to the currently predominant focus on individual behaviour change mechanisms.

### Understanding interventions as events in complex systems

Environmental interventions to promote physical activity change aspects of the circumstances in which people live and the environments that shape those circumstances. In theme 2, it was apparent that the circumstances in which people find themselves, such as the relative safety for walking or cycling in an area, are likely related to the physical structure of the environments they live in as well as conditions which are socially constructed and based on actions, perceptions, and interpretations. In theme 3 it was clear that context might also alter the success of the intervention. Although many authors reported the notion that social and physical structures interact with individual agency to influence behaviour^S32, 35, 53^, only a few described how agents and changes in structures were causally-related in the context of a system.

Authors described non-linear pathways and the “simultaneous operation of processes [...] and a web of conditions […] which involved multiple interrelationships” (p.137)^S24^, and suggested that factors were operating individually or collectively as part of a system of structural and social processes^S19, 46^. Hawe and colleagues also highlighted how systems are not conceived simply as aggregating up^S41^ and this was echoed by Lewin, who suggested that “studying groups and the ‘whole’ is fundamentally different from studying individuals: the whole is different from the sum of its parts” (p.885)^S57^.

Many authors gave examples of potential feedbacks and reciprocal pathways relating to the social environment^S23, 32, 52, 76^. For example, social norms may be established through the process of social regulation whereby behaviours change and norms are reinforced^S23, 46^. Examples of potential reinforcing mechanisms included characteristics of the built environment such as the quality of public spaces affecting the nature of social interactions within a neighbourhood, or people using environments for activity and thereby realising how safe or unsafe it is^S23, 90^.

Time was seen to be particularly important as individuals adapt to changes in environments and to others’ behaviour. Results from evaluative studies also suggested that the effects of interventions might take time to emerge^S48^. For example, studies of a new cycle hire scheme [29] and new walking and cycling routes [30] found no evidence of an effect after the first year, but significant effects in subsequent years. Other authors highlighted how systems science approaches could be used to understand the impacts of interventions in the longer term^S8, 23, 53^. Systems science suggests that system-level change is only semi-predictable and the outcomes are emergent. When viewed through a systems science lens, the potency of an environmental change arises not from the method of intervention as such, but rather from how it acts as an event that interrupts the current working of a system to create change over time^S41^. A deeper consideration of the function of the intervention (how it works to change behaviour e.g. improving the connectivity, safety or pleasantness of an area for walking and cycling) rather than describing its content (the method used or the way in which the intervention was delivered e.g. new street lights, on-road cycle lanes or off-road cycle paths)^S41^ might one way forward. It might also be one way for researchers to finding common ground between disciplines.

Authors highlighted the complexity of behaviours, interventions, and the systems in which these operate. Laitakari and Miilunpalo suggested that the triggers for physical activity are hidden from sight and comprise “extremely complex chains of acts” (p.49)^S56^. As a result, complexity might also be inherent in the social phenomenon of engaging in physical activity and in the emergence of changes in population levels of physical activity. Medical Research Council (MRC) guidance suggests that interventions may also be complex in terms of the number of interactions, the levels targeted by the intervention, the causal pathway or the variability of outcomes^S20^. Environmental changes which may act to promote physical activity often have many of these characteristics of complexity. For example, they often contain several ‘components’, whether at a small scale (e.g. a new cycle path that entails providing not only a new route as such, but also lighting, crossings and so forth) or at area level (e.g. an urban regeneration initiative that includes multiple environmental changes such as improvements to green spaces, sports facilities and signage). The causal pathways may also be long and non-linear^S76^. In general, authors of included sources did not provide a detailed description of how systems thinking could be applied to understanding how interventions work, but suggested that data could be collected and agent-based simulation models developed and tested^S8, 23, 53^.

#### Implications

Physical activity is influenced by psychological, behavioural, social, economic and physical environmental factors. These factors may be shaped by many different actors and operate at many different levels, and the casual relationships between them are often poorly understood. Because of this complexity, it is important to understand the system into which any environmental changes are introduced, and the effects of such interventions on this system. As we saw in themes 2 and 4, such interventions may interact positively or negatively with the wider system itself; for example, the effectiveness of environmental changes to promote activity may be moderated by context or concurrent influences. Those conducting evaluations should seek to describe the complexity of interventions including their different components and the system in which they are implemented^S41^. Even though some interventions may appear simple, they act upon circumstances that facilitate activity and are therefore socially embedded. Given that systems are only semi-predictable, it is unlikely that a single method, approach to analysis, or perspective on the problem will be sufficient. For example, quantitative methods which focus on measuring outcomes and explaining or predicting them in a statistical sense might be complemented by qualitative approaches to exploring, discovering and constructing explanations for differences. As a result, those conducting evaluations should consider using a variety of study designs and methods and comparing the results of different analyses [12]. These may provide valuable complementary information about different parts of the intervention or system, which together may enable more meaningful inferences to be drawn. This is not to say that evaluators should necessarily seek a detailed description of the workings of the entire social system, as this is clearly unfeasible and may in some cases be unhelpful, but they should make their perspective clear and acknowledge alternative perspectives and potential explanations. They should also be aware that changes in outcomes may take time to emerge from complex systems, and accordingly seek to collect data at appropriate times before, during and after the implementation of interventions.

### Reflections on the themes

Many of the themes were inter-related and were discussed by multiple authors to greater or lesser extents. Like others, we noted that few evaluative studies had clearly articulated the ways in which environmental changes act to promote physical activity, and some had called for greater consideration of the more generalisable ways in which environmental interventions might work to change behaviour [11, 20]. In response to these calls, we now articulate a framework of higher-order and more generalisable mechanisms that link environmental change and behaviour change, embracing the insights discussed above.

### A more general framework for intervention research

**Fig. 1:**
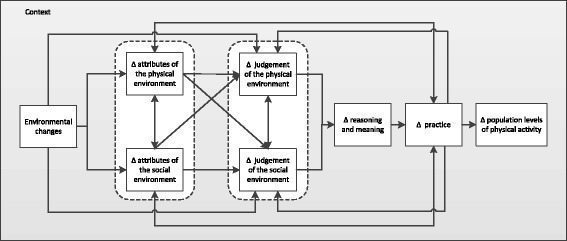
Overarching framework of potential generalisable causal pathways between environmental changes and physical activity. Dotted lines encompass sets of constructs that may be grouped or linked. Δ denotes change.

Our framework (Fig. 1) embrace the insights discussed and is intended to be flexible enough to be used by researchers from different disciplines in conceptualising and designing future intervention studies. It frames physical activity in its broadest sense rather than focussing on a narrowly defined outcome, and in terms of changes in population levels of activity. ‘Context’ forms an important and flexibly conceived backdrop and can be used, if required, to illustrate how causal pathways may be interrupted or diverge depending on the individual, physical or societal context.

To illustrate the utility of the framework, let us follow the example of the introduction of a new path for walking and cycling. Although this may appear to be a single intervention, it is likely to involve changing a range of specific environmental attributes such as the connectivity of the area, the directness of routes between destinations, the separation of bicycles from motor vehicles or the amount of lighting. These changes are observable and may be quantifiable. Changes in attributes of the social environments, such as social support and social cohesion, may also be instigated, and some of these may also be observed or inferred (e.g. through changes in the nature of interactions between pedestrians and cyclists). Changes in attributes of the physical and social environment may lead to changes in people’s (conscious or unconscious) judgements and reasoning. For example, improved segregation and lighting may be judged as creating a safer environment in which users are less likely to be involved in an accident. These changes in judgements are socially constructed and based in part on an individual’s assessment of how other people use the space, for example whether pedestrians and cyclists use the path together and how they interact. Judgements may also be formed from historical contexts and social meanings about the place, its former use, and whether it is now acceptable to use it. It is therefore unlikely that any such judgements will be related solely to ‘physical’ or ‘social’ attributes; instead, they are likely to lie on a continuum between these reciprocal domains of influence or to involve both. There may then be a change in reasoning or meaning concerning the potential acceptability or logic of a practice consequent on, for example, a change in the risk of engaging in walking. Changes in reasoning may be conscious, but changes in meaning ascribed to practice might be conscious or unconscious. Mechanisms or outcomes may or may not be activated, depending on the context: changes in reasoning about use of the path might vary according to individual context, such as a person’s beliefs about walking and its merits, but also according to other social or geographic contexts, such as climate. The framework depicts this interaction between context and mechanisms by including context as the backdrop. If a change in practice occurs in a sufficient proportion of the population, this may give rise to changes in population levels of physical activity. A comprehensive evaluation might therefore aim to collect data on frequency of use of the infrastructure, total levels of walking or cycling (both for transport or recreation), total physical activity (among other things), and might use qualitative or quantitative data about beliefs surrounding walking and cycling, the cultural and historical use of the space, perceptions of the environment including safety or pleasantness, why and how people use the infrastructure to understand differential, unexpected or absent effects in certain groups.

An alternative example of an environmental intervention is that of an urban regeneration or neighbourhood renewal initiative [31]. Such interventions comprise a package of measures that may include improvements to existing homes, the demolition of other homes and construction of new ones, improvements to the physical environment of the neighbourhood, new or improved amenities and services, and community programmes. Just as in the previous example of a new path for walking or cycling, these may change several attributes such as the physical qualities of the area through improved lighting, access to greenspace, the provision of shops and services and the level of community activity and support in a neighbourhood. The extent to which changes in environmental attributes such as aesthetic appeal, accessibility of amenities or antisocial behaviour are considered ‘social’ or ‘physical’ might depend on both the epistemological perspective and the local context (if, for example, a new sports facility is perceived as not primarily intended for local residents [22]).

### Strengths and limitations of the review

Our review has a number of strengths and limitations. We have transparently described the process of searching for and synthesising evidence in line with guidelines for thematic and narrative synthesis [13, 14]. We used a limited set of search terms, seeking an efficient balance between the breadth of the search (sensitivity) and the number of results (specificity). Newer relevant material may have been missed, but we did not intend to present a comprehensive assessment of all available literature, seeking rather to understand the range of conceptual issues discussed. We did not elicit additional (unpublished) reflections from researchers from different disciplines, which might have provided a degree of validation of our analysis. However, in our experience of working in multidisciplinary teams including those from public health, geography, transport, psychology, and social sciences more generally, we believe that our synthesis reflects the breadth of conceptual perspectives applied or relevant to this field. Neither did we seek to critique existing theoretical models or quantify their use in previous research. Instead, and in keeping with our search strategy, we used thematic interpretative synthesis to contrast different types of research material from different disciplines, acknowledging their differing epistemological provenances and exploring areas of similarity and dissonance. We did not aim to judge conceptualisations as ‘right’ or ‘wrong’, but rather to investigate the different approaches used and the implications of each. There is no single ‘correct’ way of synthesising heterogeneous bodies of quantitative and qualitative literature [23], but we believe that our approach - albeit clearly reflecting our own particular disciplinary and other interpretations - is grounded in the evidence and has enabled us to produce collective meaning from the disparate material at our disposal. From this synthesis we elicited high-level themes and used these to draw out generalisable mechanisms relevant to how a broad range of environmental interventions might promote physical activity, an area of theory that has been poorly articulated in the literature to date [11, 20]. We were unable to provide summaries of the advantages and disadvantages of all approaches; for that information, we direct readers to the more detailed sources cited in the review. We hope our work will stimulate further discussion among researchers from different disciplines, will help evaluators to frame and design future intervention research in this field, and will help commissioners, funders and users of evaluations to reflect on the relevant mechanisms and interpret results. Although we focused on the external physical environment, aspects of our review may also be relevant to interventions in the internal built environment.

## Conclusions

We have synthesised evidence from multiple disciplines ranging from economics, sociology and psychology to public health and urban design and applied the insights to external physical environmental changes which may promote physical activity. We have highlighted similarities in basic scientific understanding between disciplines, and the ways in which common methodological ground might be found in this field of intervention research in spite of other differences. Rigorous and thoughtful evaluations of environmental and policy interventions are required in which the outcomes are appropriately matched to the intervention and the putative mechanisms of the intervention have been considered. Evaluators should at least acknowledge the philosophical perspective adopted, and take account of the ways and social systems in which physical activity is undertaken. Studies should seek to understand how interventions work through considering these systems, the context in which interventions take place, and the (un) observable mechanisms that may operate using a combination of qualitative and quantitative methods. Results will then be more easily interpreted and applied by policymakers. Our framework describes a general set of causal pathways which can applied to a range of types of environmental intervention, and is flexible enough to be used by researchers from different disciplines in conceptualising and designing these studies.

## Additional file

Additional file 1: List of included papers. (DOCX 28 kb)

## Abbreviations

MRC: Medical research council
NICE: National institute for health and care excellence
