# Supplementary material for: Physical activity and the environment: conceptual review and framework for intervention research
Source: Int J Behav Nutr Phys Act. 2017 Nov 15;14:156. doi: 10.1186/s12966-017-0610-z (PMC5688667; doi:10.1186/s12966-017-0610-z)
Supplement: Additional file 1: — List of included papers. (DOCX 28 kb) [file 12966_2017_610_MOESM1_ESM.docx]

**Additional file 1: List of included papers**

S1. Abraham, A., Sommerhalder, K., and Abel, T., Landscape and well-being: a scoping study on the health-promoting impact of outdoor environments. International Journal of Public Health, 2010. 55(1): p. 59-69.

S2. Adler, N.E. and Stewart, J., Reducing obesity: motivating action while not blaming the victim. Milbank Quarterly, 2009. 87(1): p. 49-70.

S3. Ajzen, I., The theory of planned behavior. Organizational Behavior and Human Decision Processes, 1991. 50(2): p. 179-211.

S4. Alfonzo, M.A., To walk or not to walk? The hierarchy of walking needs. Environment and Behavior, 2005. 37(6): p. 808-836.

S5. Annear, M., et al., Environmental influences on healthy and active ageing: a systematic review. Ageing & Society, 2014. 34(4): p. 590-622.

S6. Anonymous, Increasing physical activity. A report on recommendations of the Task Force on Community Preventive Services. Morbidity & Mortality Weekly Report. Recommendations & Reports, 2001. 50(RR-18): p. 1-14.

S7. Astbury, B. and Leeuw, F.L., Unpacking black boxes: mechanisms and theory building in evaluation. American Journal of Evaluation, 2010. 31(3): p. 363-381.

S8. Backholer, K., et al., A framework for evaluating the impact of obesity prevention strategies on socioeconomic inequalities in weight. American Journal of Public Health, 2014. 104(10): p. e43-50.

S9. Baker Philip, R.A., et al. Community wide interventions for increasing physical activity (review). Cochrane Database of Systematic Reviews, 2015. DOI: 10.1002/14651858.CD008366.pub3.

S10. Bandura, A., Social foundations of thought and action: A social cognitive theory. Social learning theory. 1986, Englewood Cliffs, NJ, US: Prentice-Hall, 617.

S11. Bargh, J. and Chartrand, T., The unbearable automaticity of being. American Psychologist, 1999. 54: p. 462 - 479.

S12. Bauman, A.E., et al., Toward a better understanding of the influences on physical activity: The role of determinants, correlates, causal variables, mediators, moderators, and confounders. American Journal of Preventive Medicine, 2002. 23(2, Supplement 1): p. 5-14.

S13. Bedimo-Rung, A.L., Mowen, A.J., and Cohen, D.A., The significance of parks to physical activity and public health: a conceptual model. American Journal of Preventive Medicine, 2005. 28(2): 159-68.

S14. Blacksher, E. and Lovasi, G.S., Place-focused physical activity research, human agency, and social justice in public health: taking agency seriously in studies of the built environment. Health & Place, 2012. 18(2): p. 172-9.

S15. Bordieu, P., The logic of practice (translated in 1990 by Nice R). 1980, Stanford, California: Stanford University Press.

S16. Boyce, C., Walkability, social inclusion and social isolation and street redesign. Built environment 2010. 36(4): p. 461-73.

S17. Brennan Ramirez, L.K., et al., Indicators of activity-friendly communities: an evidence-based consensus process. American Journal of Preventive Medicine, 2006. 31(6): p. 515-24.

S18. Brownson, R.C., Haire-Joshu, D., and Luke, D.A., Shaping the context of health: a review of environmental and policy approaches in the prevention of chronic diseases. Annual Review of Public Health, 2006. 27: p. 341-70.

S19. Carlson, C., et al., Complexity in built environment, health, and destination walking: a neighborhood-scale analysis. Journal of Urban Health, 2012. 89(2): p. 270-84.

S20. Craig, P., et al., Developing and evaluating complex interventions: the new Medical Research Council guidance. BMJ, 2008. 337.

S21. Cunningham, G.O. and Michael, Y.L., Concepts guiding the study of the impact of the built environment on physical activity for older adults: a review of the literature. American Journal of Health Promotion, 2004. 18(6): p. 435-43.

S22. Dalkin, S.M., et al., What’s in a mechanism? Development of a key concept in realist evaluation. Implementation Science, 2015. 10(1): p. 49.

S23. Diez Roux, A.V., Residential environments and cardiovascular risk. Journal of Urban Health, 2003. 80(4): p. 569-89.

S24. Diez Roux, A.V. and Mair, C., Neighborhoods and health, in Biology of Disadvantage: Socioeconomic Status and Health, Adler NE and Stewart, J. 2010, Wiley-Blackwell: Malden. p. 125-145.

S25. Ding, D. and Gebel, K., Built environment, physical activity, and obesity: what have we learned from reviewing the literature? Health & Place, 2012. 18(1): p. 100-5.

S26. Dunn, A.L., Getting started--a review of physical activity adoption studies. British Journal of Sports Medicine, 1996. 30(3): p. 193-9.

S27. Dunton, G.F., Cousineau, M., and Reynolds, K.D., The intersection of public policy and health behavior theory in the physical activity arena. Journal of Physical Activity & Health, 2010. 7 Suppl 1: p. S91-8.

S28. Engbers, L.H., et al., Worksite health promotion programs with environmental changes: a systematic review. American Journal of Preventive Medicine, 2005. 29(1): p. 61-70.

S29. Epstein, L.H., Integrating theoretical approaches to promote physical activity. American Journal of Preventive Medicine, 1998. 15(4): p. 257-65.

S30. Fogelholm, M. and Lahti-Koski, M., Community health-promotion interventions with physical activity: Does this approach prevent obesity? Scandinavian Journal of Nutrition/Naringsforskning, 2002. 46(4): p. 173-177.

S31. Foster C, et al., Understanding Participation in Sport: A systematic review. 2005, Sport England: London.

S32. Foster, C. and Hillsdon, M., Changing the environment to promote health-enhancing physical activity. Journal of Sports Sciences, 2004. 22(8): p. 755-69.

S33. Frohlich, K.L., Corin, E., and Potvin, L., A theoretical proposal for the relationship between context and disease. Sociology of Health & Illness, 2001. 23(6): p. 776-797.

S34. Giddens, A., The constitution of society: outline of theory of structuration. 1984, University of California Press: Los Angeles. p. 25.

S35. Giles-Corti, B., People or places: what should be the target? Journal of Science & Medicine in Sport, 2006. 9(5): p. 357-66.

S36. Giles-Corti, B. and King, A.C., Creating active environments across the life course: "thinking outside the square". British Journal of Sports Medicine, 2009. 43(2): p. 109-13.

S37. Giles-Corti, B., et al., Understanding physical activity environmental correlates: increased specificity for ecological models. Exercise & Sport Sciences Reviews, 2005. 33(4): p. 175-81.

S38. Golden, S.D. and Earp, J.A., Social ecological approaches to individuals and their contexts: twenty years of health education & behavior health promotion interventions. Health Education & Behavior, 2012. 39(3): p. 364-72.

S39. Handy, S.L., et al., How the built environment affects physical activity: views from urban planning. American Journal of Preventive Medicine, 2002. 23(2 Suppl): p. 64-73.

S40. Harris, J.K., et al., Mapping the development of research on physical activity and the built environment. Preventive Medicine, 2013. 57(5): p. 533-40.

S41. Hawe, P., Shiell, A., and Riley, T., Theorising Interventions as Events in Systems. American Journal of Community Psychology, 2009. 43(3-4): p. 267-276.

S42. Heath, G.W., et al., The effectiveness of urban design and land use and transport policies and practices to increase physical activity: A systematic review. Journal of Physical Activity and Health, 2006. 3(Suppl 1): p. S55-S76.

S43. Hoehner, C.M., et al., Opportunities for integrating public health and urban planning approaches to promote active community environments. American Journal of Health Promotion, 2003. 18(1): p. 14-20.

S44. Hollands, G.J., et al., Altering micro-environments to change population health behaviour: towards an evidence base for choice architecture interventions. BMC Public Health, 2013. 13: p. 1218.

S45. Horne, M. and Tierney, S., What are the barriers and facilitators to exercise and physical activity uptake and adherence among South Asian older adults: a systematic review of qualitative studies. Preventive Medicine, 2012. 55(4): p. 276-84.

S46. Huie, S.A.B., The concept of neighborhood in health and mortality research. Sociological Spectrum, 2001. 21(3): p. 341-358.

S47. Humphreys, D.K. and Ogilvie, D., Synthesising evidence for equity impacts of population-based physical activity interventions: a pilot study. International Journal of Behavioral Nutrition & Physical Activity, 2013. 10: p. 76.

S48. Hunter, R.F., et al., The impact of interventions to promote physical activity in urban green space: a systematic review and recommendations for future research. Soc Sci Med, 2015. 124: p. 246-56.

S49. Ickes, M.J. and Sharma, M., A systematic review of physical activity interventions in Hispanic adults. Journal Of Environmental & Public Health, 2012. article id.: 156435, pages 1-15.

S50. Kahn, E.B., et al., The effectiveness of interventions to increase physical activity. A systematic review. American Journal of Preventive Medicine, 2002. 22(4 Suppl): p. 73-107.

S51. King, A.C., How to promote physical activity in a community: research experiences from the US highlighting different community approaches. Patient Education & Counseling, 1998. 33(1 Suppl): p. S3-12.

S52. King, A.C., et al., Multilevel modeling of walking behavior: advances in understanding the interactions of people, place, and time. Medicine & Science in Sports & Exercise, 2008. 40(7 Suppl): p. S584-93.

S53. King, A.C., et al., Theoretical approaches to the promotion of physical activity: forging a transdisciplinary paradigm. American Journal of Preventive Medicine, 2002. 23(2 Suppl): p. 15-25.

S54. Kirk, S.F., Penney, T.L., and McHugh, T.L., Characterizing the obesogenic environment: the state of the evidence with directions for future research. Obesity Reviews, 2010. 11(2): p. 109-17.

S55. Kremers, S.P., et al., Environmental influences on energy balance-related behaviors: A dual-process view. International Journal of Behavioral Nutrition and Physical Activity, 2006. 3(1): p. 9.

S56. Laitakari, J. and Miilunpalo, S., How can physical activity be changed--basic concepts and general principles in the promotion of health-related physical activity. Patient Education & Counseling, 1998. 33(1 Suppl): p. S47-59.

S57. Lewin, K., Field Theory and Experiment in Social Psychology: Concepts and Methods. American Journal of Sociology, 1939. 44(6): p. 868-896.

S58. Loukaitou-Sideris, A. and Eck, J.E., Crime prevention and active living. American Journal of Health Promotion, 2007. 21(4 Suppl): p. 380-9, iii.

S59. Macintyre, S., Ellaway, A., and Cummins, S., Place effects on health: how can we conceptualise, operationalise and measure them? Soc Sci Med, 2002. 55(1): p. 125-39.

S60. Matson-Koffman, D.M., et al., A site-specific literature review of policy and environmental interventions that promote physical activity and nutrition for cardiovascular health: what works? American Journal of Health Promotion, 2005. 19(3): p. 167-93.

S61. Mayne, S.L., Auchincloss, A.H., and Michael, Y.L., Impact of policy and built environment changes on obesity-related outcomes: a systematic review of naturally occurring experiments. Obesity Reviews, 2015. 16(5): p. 362-75.

S62. McCormack, G.R. and Shiell, A., In search of causality: a systematic review of the relationship between the built environment and physical activity among adults. International Journal of Behavioral Nutrition & Physical Activity, 2011. 8: p. 125.

S63. McLeroy, K.R., et al., Social socience theory in health education: time for a new model? Health Education Research, 1993. 8(3): p. 305-312.

S64. McNeill, L.H., Kreuter, M.W., and Subramanian, S.V., Social environment and physical activity: a review of concepts and evidence. Social Science & Medicine, 2006. 63(4): p. 1011-22.

S65. Michie, S., van Stralen, M., and West, R., The behaviour change wheel: A new method for characterising and designing behaviour change interventions. Implementation Science, 2011. 6(1): p. 42.

S66. Mozaffarian, D., et al., Population approaches to improve diet, physical activity, and smoking habits: a scientific statement from the American Heart Association. Circulation, 2012. 126(12): p. 1514-63.

S67. National Institute for Health and Care Excellence, Four commonly used methods to increase physical activity. 2006.

S68. National Institute for Health and Care Excellence, A rapid review of the effectiveness of community-based walking and cycling programmes to promote physical activity in adults.2006; London, UK: National Institute for Health and Care Excellence.

S69. National Institute for Health and Care Excellence, Physical activity and the environment. 2008; London, UK: National Institute for Health and Care Excellence.

S70. National Institute for Health and Care Excellence, Promoting physical activity in the workplace. 2008; London, UK: National Institute for Health and Care Excellence.

S71. National Institute for Health and Care Excellence, Preventing type 2 diabetes: population and community-level interventions. 2011; London, UK: National Institute for Health and Care Excellence.

S72. National Institute for Health and Care Excellence, Walking and cycling: local measures to promote walking and cycling as forms of travel or recreation. 2012; London, UK: National Institute for Health and Care Excellence.

S73. National Institute for Health and Care Excellence, Preventing type 2 diabetes : population and community-level interventions. Evidence Update; 2014. London, UK: National Institute for Health and Care Excellence; 2014.

S74. National Institute for Health and Care Excellence, Physical activity and the environment : Evidence Update; 2014. London, UK: National Institute for Health and Care Excellence.

S75. Nettleton, S. and Green, J., Thinking about changing mobility practices: how a social practice approach can help. Sociology of Health & Illness, 2014. 36(2): p. 239-51.

S76. Ogilvie, D., et al., An applied ecological framework for evaluating infrastructure to promote walking and cycling: the iConnect study. American Journal of Public Health, 2011. 101(3): p. 473-81.

S77. Olsen, JS3.M., An integrative review of literature on the determinants of physical activity among rural women. Public Health Nursing, 2013. 30(4): p. 288-311.

S78. Owen, N., et al., Understanding environmental influences on walking; Review and research agenda. American Journal of Preventive Medicine, 2004. 27(1): p. 67-76.

S79. Pikora, T., et al., Developing a framework for assessment of the environmental determinants of walking and cycling. Social Science & Medicine, 2003. 56(8): p. 1693-703.

S80. Plotnikoff, R.C. and Karunamuni, N., Steps towards permanently increasing physical activity in the population. Current Opinion in Psychiatry, 2011. 24(2): p. 162-167.

S81. Pope, C., Mays, N., and Popay, J., Synthesizing qualitative and quantitative health evidence: a guide to methods 2007, New York: Open University Press.

S82. Pratt, C.A., et al., Design characteristics of worksite environmental interventions for obesity prevention. Obesity, 2007. 15(9): p. 2171-80.

S83. Reid, R.D., Why don't they stay changed? Canadian Journal of Cardiology, 1995. 11 Suppl A: p. 26A-29A.

S84. Rissel, C. and Garrard, J., Cycling for Active Transport and Recreation in Australia: Status Review and Future Directions. World Transport Policy & Practice, 2006. 13(1): p. pp 49-63.

S85. Russell, J., The success of worksite nutrition and physical activity interventions with environmental modifications A systematic review. 2012, The University of Texas School of Public Health Dissertation: AAI1515654.

S86. Rutten, A. and Gelius, P., The interplay of structure and agency in health promotion: integrating a concept of structural change and the policy dimension into a multi-level model and applying it to health promotion principles and practice. Social Science & Medicine, 2011. 73(7): p. 953-9.

S87. Rychetnik, L., et al., Criteria for evaluating evidence on public health interventions. Journal of Epidemiology and Community Health, 2002. 56(2): p. 119-127.

S88. Sallis, J.F., Bauman, A., and Pratt, M., Environmental and policy interventions to promote physical activity. American Journal of Preventive Medicine, 1998. 15(4): p. 379-97.

S89. Sallis, J.F., et al., An ecological approach to creating active living communities. Annual Review of Public Health, 2006. 27: p. 297-322.

S90. Schulz, A.J., et al., Healthy eating and exercising to reduce diabetes: Exploring the potential of social determinants of health frameworks within the context of community-based participatory diabetes prevention. American Journal of Public Health, 2005. 95(4): p. 645-651.

S91. Shove, E., Pantzar, M., and Watson, M., The Dynamics of Social Practice 2012, London: Sage.

S92. Siddiqi, Z., Tiro, J.A., and Shuval, K., Understanding impediments and enablers to physical activity among African American adults: a systematic review of qualitative studies. Health Education Research, 2011. 26(6): p. 1010-24.

S93. Spence, J.C. and Lee, R.E., Toward a comprehensive model of physical activity. Psychology of Sport and Exercise, 2003. 4(1): p. 7-24.

S94. Stafford, M., et al., Pathways to obesity: identifying local, modifiable determinants of physical activity and diet. Social Science & Medicine, 2007. 65(9): p. 1882-97.

S95. Stewart, G., Anokye, N.K., and Pokhrel, S., What interventions increase commuter cycling? A systematic review. BMJ Open, 2015. 5(8): p. e007945.

S96. Stokols, D., Translating social ecological theory into guidelines for community health promotion. Am J Health Promot, 1996. 10(4): p. 282-98.

S97. Sugiyama, T. and Ward-Thompson, C., Outdoor environments, activity and the well-being of older people; conceptualising environmental support. ENVIRON & PLANN A, 2007. 39(8): p. 1943-60.

S98. Swinburn, B., Egger, G., and Raza, F., Dissecting obesogenic environments: the development and application of a framework for identifying and prioritizing environmental interventions for obesity. Preventive Medicine, 1999. 29(6 Pt 1): p. 563-70.

S99. To, Q.G., et al., Workplace physical activity interventions: a systematic review. American Journal of Health Promotion, 2013. 27(6): p. e113-23.

S100. Townshend, T. and Lake, A.A., Obesogenic urban form: Theory, policy and practice. Health & Place, 2009. 15(4): p. 909-916.

S101. Watts, P., et al., The influence of environmental factors on the generalisability of public health research evidence: physical activity as a worked example. International Journal of Behavioral Nutrition & Physical Activity, 2011. 8: p. 128.

S102. Wilson, D.K., Commentary for Health Psychology special issue: theoretical advances in diet and physical activity interventions. Health Psychology, 2008. 27(1 Suppl): p. S1-2.

S103. World Health Organisation, A guide for population-based approaches to increasing levels of physical activity: implementation of the WHO global strategy on diet, physical activity and health, World Health Organisation, Editor. 2007: Geneva.

S104. Yancey, A.K., et al., Population-based interventions engaging communities of color in healthy eating and active living: a review. Preventing Chronic Disease, 2004. 1(1): p. A09.
